# Unveiling, Analyzing the Mechanisms of, and Proposing Solutions for Bribery in Japan's Medical Device Sector

**DOI:** 10.7759/cureus.61285

**Published:** 2024-05-29

**Authors:** Akihiko Ozaki, James Larkin, Natsuya Sakata, Yudai Kaneda, Erika Yamashita, Hiroaki Saito, Tetsuya Tanimoto, Mihajlo Jakovljevic

**Affiliations:** 1 Breast and Thyroid Surgery, Jyoban Hospital of Tokiwa Foundation, Iwaki, JPN; 2 Department of General Practice, Royal College of Surgeons in Ireland University of Medicine and Health Sciences, Dublin, IRL; 3 School of Medicine, Akita University, Akita, JPN; 4 School of Medicine, Hokkaido University, Sapporo, JPN; 5 Healthcare, Medical Governance Research Institute, Tokyo, JPN; 6 Gastroenterology, Soma Central Hospital, Soma, JPN; 7 Internal Medicine, Navitas Clinic, Tokyo, JPN; 8 Institute of Comparative Economic Studies, Hosei University, Tokyo, JPN

**Keywords:** healthcare professionals, post-marketing surveillance, japan, transparency, medical devices

## Abstract

Both public and academic scrutiny of the financial relationships between the medical device industry and the healthcare society occur less frequently than those involving the pharmaceutical industry, and Japan is no exception to these shortcomings. This paper examines the ethical and legal challenges inherent in Japan's medical device industry through the lens of bribery scandals, placing these issues within the broader context of global healthcare corruption.

It aims to derive lessons and suggest universal strategies for ethical and legal enhancements. The discussion includes two notable cases: one involving inappropriate transactions between a cancer center and a biliary stent manufacturer, and another concerning a corrupt donation scheme between a medical device company and a university's anesthesiology department, which was found guilty. In our analysis, we also acknowledge the industry's efforts toward compliance and reform to maintain a balanced perspective.

The analysis not only highlights the unique culture and structure of the Japanese medical device industry, such as the exploitation of flexible pricing and opaque financial practices but also contrasts these issues with the tightly regulated pharmaceutical industry. This approach reveals both sector-specific challenges and common corruption drivers, enhancing our understanding of why such scandals occur and persist.

We propose ethical and compliance-focused business measures such as centralizing donation decisions, limiting the financial independence of marketing divisions, and increasing transparency, alongside adopting mandatory disclosure practices based on successful models from the United States and Europe. By emphasizing integrity and presenting diverse perspectives, this study aims to elevate ethical and legal standards in the medical device industry and improve patient health outcomes worldwide.

## Editorial

Medical devices are characterized as instruments and equipment for application in the diagnosis, treatment, or prevention of diseases affecting humans or animals [1]. Examples of medical devices include tongue depressors, rapid antigen tests, and joint replacements. In the medical device industry, promotional activities aimed at stimulating sales are conducted with as much zeal as in the pharmaceutical industry [2,3]. Such promotions can commonly lead to conflicts of interest that potentially influence medical practices and may further result in various scandals, exerting serious impacts on society [4]. For example, Annapureddy et al. used the United States Open Payments Database of financial relationships between medical device manufacturers and doctors to demonstrate that patients were more likely to receive an implantable cardioverter-defibrillator or cardiac resynchronization therapy-defibrillator from the manufacturer that made the largest payments to their doctors when compared to three other manufacturers [4]. This illustrates that, beyond the current scandal, the financial relationships between medical device manufacturers and healthcare professionals represent a broader issue.

However, at least in Japan, the effects of these conflicts of interest and scandals have predominantly been issues for the pharmaceutical industry, with the majority of reporting and research focusing on them [5-7]. As a result, improvements are being observed in the relationships between the pharmaceutical industry and the healthcare society [8], whereas sufficient scrutiny has not been applied to the medical device industry. This backdrop suggests that, in Japan, pre-modern practices, not observed in the pharmaceutical industry, may still be occurring within the medical device industry, even though the country has a large market for medical devices, valued at 4.41 trillion Japanese yens (JPY) (approximately 28 billion United States dollars (USD, based on the exchange rate of 156.78 JPY/USD on May 28, 2024).

It is noteworthy that, in the past few years, a series of bribery incidents have emerged, entailing illicit financial transactions between the medical device industry and healthcare professionals in Japan [9]. Although the underlying mechanisms are multifaceted, it has been observed that under-the-table payments often play a significant role in the promotional strategies employed within the medical community by the Japanese medical device industry. Additionally, the medical device industry's ethical and legal challenges are not new to Japan, as Japan's medical device industry has had instances ranging from safety concealments to anti-competitive practices, prompting decades of reforms and a push for integrity, despite ongoing issues with emerging technologies [10-12]. Furthermore, with recent advancements in artificial intelligence and the development of medical devices that utilize it [13,14], an increasing number of new companies are entering the market. This influx could intensify market competitiveness and lead to more aggressive marketing and promotional efforts, primarily targeting healthcare professionals and organizations. In this context, it is now the ideal time to discuss the ethical and legal aspects of medical device marketing from various standpoints.

This paper explores two recent bribery cases highlighted by the Japanese nationwide mass media using the data and information available on the Internet. Both cases underwent legal scrutiny, and in one of the cases, the actions were conclusively determined to be illegal. These cases will help elucidate the underlying dynamics and allow us to propose actionable strategies to prevent such unethical practices.

Case one: National Cancer Center East Hospital, Kashiwa, Japan, and Zeon Medical Inc., Tokyo, Japan

In September 2023, a bribery case involving a selection of medical devices concerning biliary stents at the National Cancer Centre East Hospital, one of the most influential cancer centers in Japan, came to light [15]. A doctor who had previously worked at the Department of Hepatobiliary and Pancreatic Oncology at the hospital and a former president of Zeon Medical, a manufacturer of the devices, were arrested for bribery [15]. It is reported that the doctor received 10,000 JPY (63 USD) per stent for nominal cooperation in the post-market surveillance research into the usability of the products, but it was divulged that no such research was actually conducted. In the fiscal year 2020, the doctor reportedly used about 150 stents manufactured by Zeon Medical [15]. Given its recent occurrence, details about this case remain limited. As of May 23, 2024, further updates on the incident are not available on the internet.

Case two: Clinical Anesthesia Department of Mie University, Tsu, Japan, and Nihon Kohden Corporation, Tokyo, Japan

In January 2023, a guilty verdict was issued by the Tsu District Court against Nihon Kohden Corporation, a Japanese medical device company, for bribery [16]. A series of scandals were reported at the Clinical Anesthesia Department of Mie University in Japan. In this case, the company made a donation of 2 million JPY (13 thousand USD) to a third-party organization, which the former professor of the Department of Clinical Anesthesia at Mie University was director of. The professor, who oversaw the committee responsible for selecting operating theatre equipment, favored the company that provided a donation by helping it secure the rights to replace the existing equipment. This quid pro quo arrangement was judged as an act of bribery. It was revealed that the donation bypassed the formal approval processes typically required by Nihon Kohden Corporation [16]. To facilitate this, local company representatives initially sold the operating theatre equipment to a wholesale company at steep discounts, allowing the wholesaler to realize substantial profits. These profits were then used by the wholesaler to make a bribe to the professor's institution, effectively using the falsely generated profit margin as the funding source.

Mechanisms of the scandals

In case one, the concern arose from the potential for post-marketing surveillance to become a hotbed for fraud. Post-marketing surveillance is supposed to involve the collection of safety information using real-world data. However, it can also be utilized for promotional purposes. In the case at hand, it was used for promotion, and moreover, payments were made to individual doctors without any actual surveillance being conducted. Pharmaceutical companies have been alleged to have engaged in similar practices [17]. It is necessary to be vigilant about the risk that this system could lead to a financial scandal as shown in this case.

A critical issue highlighted in case two is the concept of under-the-table payment by the Nihon Kohden Corporation. In Japan, there is considerable flexibility in the pricing of medical devices, unlike the stringent regulation of drug prices. Manufacturers of medical devices, such as Nihon Kohden Corporation, have the discretion to apply discounts at will, in stark contrast to the pharmaceutical industry [18]. In the pharmaceutical industry, drug prices are tightly controlled by the government, and the influence of wholesale companies, which serve as intermediaries between pharmaceutical companies and healthcare professionals, is substantial. As a result, pharmaceutical companies cannot autonomously determine the prices of their drugs. This ability to freely offer discounts is a distinctive and crucial aspect of the medical device industry in Japan, which diverges markedly from the rigid price-setting practices in the pharmaceutical industry [19] and creates space for corrupt practices.

Understanding the contrasting scales of the pharmaceutical and medical device industries in Japan is crucial. The pharmaceutical industry boasts a robust market of over 10 trillion JPY (64 billion USD), concentrated among less than 100 companies within the Japan Pharmaceutical Manufacturers Association (JPMA). In comparison, the medical device industry is segmented into 106 categories and encompasses 20 professional medical organizations under the Japan Federation of Medical Devices Associations (JFMDA), which is the largest trade organization of medical device companies and their associations in the country; yet, its market size is still about 4.41 trillion JPY (28 billion USD) as explained above. This indicates a much larger number of smaller-scale companies in the medical device sector compared to the more consolidated pharmaceutical industry [20]. In fact, Zeon Medical is headquartered in central Tokyo but has only 216 employees as of April 1, 2021 [21]. Compared to pharmaceutical and other companies, it is extremely small, and partly as a result of this, governance may not have been sufficiently effective. The small size of medical device companies is also considered a barrier to improved governance outside of Japan [22].​​​​​

Solutions

To ameliorate these issues, first of all, medical device companies must adhere more strictly to existing regulations. At a minimum, decisions regarding donations to the medical community should be centralized at the corporate head office level, given a decision to make the relevant donation was not made in the headquarters of Nihon Kohden Corporation. Further, it is imperative that promotional divisions be prohibited from making autonomous decisions about such donations to prevent any discretionary impropriety. Indeed, similar transitions have been recently made in pharmaceutical industries in Japan.

Second, given that ending the practice of setting individual sales targets for marketing representatives, a practice introduced by GlaxoSmithKline plc, Brentford, United Kingdom, in 2013 (though later discontinued) [23,24], is unlikely to occur in the short term, other measures need to be considered including increasing the transparency of payments to healthcare professionals and healthcare organizations made by medical device companies. Currently, a majority of these types of payments are disclosed in accordance with the transparency guidelines issued by the JFMDA [25], a framework that is the same as that issued by JPMA [26,27]. However, multiple medical device companies do not publicly disclose their financial contributions to the medical community. While the exact motivations for this lack of transparency are unclear, a notable consideration is the organizational structure of industry associations [28]. Medical device companies are typically members of specialized associations under the broader JFMDA umbrella. For instance, manufacturers of medical endoscopes and ophthalmic devices are often part of the Japan Medical Optical Instruments Manufacturers Association. This separation may lead individual companies to believe that they are not directly obligated to adhere to the transparency rules set by the JFMDA.

These issues are not limited to Japan. For example, in order to understand the wider East Asian and Association of South‐East Asian Nations (ASEAN) countries context, one should know that legislation on medical devices marketing approval and reimbursement, unlike with pharmaceuticals, remains exceptionally diverse and heterogeneous across this vast region [29]. Furthermore, safety efficacy and quality requirements are not uniform across these jurisdictions [30]. Such a landscape creates room for different interpretations by the manufacturers and regulatory authorities [31]. These misinterpretations in legal interpretation (either deliberate or accidental) might occasionally increase the risk of fraudulent business behavior [32].

Similar issues in terms of transparency exist in Europe [33], where in some countries, only a small percentage of medical device companies have signed up for a system of self-regulated disclosure and this only applies to education-related payments [2]. On the other hand, the self-regulated disclosure system for pharmaceutical companies in most European countries, though it has limitations, covers a broader range of payments (e.g., consultancy and research and development) and includes a larger percentage of companies [22,34,35]. Several countries (e.g., the United States, France, and Portugal) have legally mandated both pharmaceutical and medical device companies to publish details of consultancy, education, and other forms of payments that have been, in some cases, used for bribery [36,37]. However, research and development payments, which are likely to include post-marketing surveillance payments, are often not included in this [38,39]. Analyses have found that mandated transparency showed a high level of payments from the medical device industry to healthcare professionals in the United States [3]. Major issues have been highlighted with the systems of self-regulation [2,34], potentially pointing to the need for mandated transparency.

In order to address potential bribery associated with post-marketing surveillance, the introduction of independent processes for post-marketing surveillance should be considered. This could address the issues described above as well as a range of other issues such as the use of post-marketing surveillance as a tool to encourage off-label prescribing [40]. These independent processes could be commissioned by the government and carried out using public funding, as is done in New Zealand, where the Medicine and Medical Devices Safety Authority now conducts some post-market surveillance through the University of Otago, Dunedin, New Zealand [41].

The underlying legal issues in this case are not easily resolved. However, in advancing transparency regarding financial relationships between the medical device industry and healthcare society, both in Japan and internationally, it is crucial to consider the broader impacts on public health and the diverse viewpoints of all stakeholders involved, namely the patients and the public. A framework should be established to regularly incorporate feedback from these stakeholders, enabling a more comprehensive understanding of the consequences of inadequate transparency. Additionally, gathering relevant data and evidence is vital. By discussing these perspectives alongside empirical data on the implications of non-disclosure for public health outcomes and healthcare costs, we can guide more informed, effective legislative and corporate policy changes that enhance accountability and trust across the healthcare society.

Conclusions

In conclusion, an examination of the two recent scandal cases in Japan’s medical device industry highlights the under-the-table practices and the disparities in pricing flexibility and company scale within the medical device industry. Despite the considerable market size and the dominance of a few large companies in the pharmaceutical sector, it can be said that it is the medical device industry, with its multitude of smaller-scale entities, that has exhibited a greater inclination towards opaque financial transactions. The recent scandals have acted as a catalyst for much-needed reforms, encouraging a move toward enhanced transparency and more rigorous adherence to regulations within and beyond Japan. Although the ultimate impact of these reforms is yet to be determined, the current trend toward transparency and ethical compliance represents an advancement for the industry, one that can be built on. It emphasizes the importance of integrity in the relationships between medical device companies and healthcare professionals, with the potential outcome of progressing medical research and improving the health of patients.
